# Anthropogenic selection enhances cancer evolution in Tasmanian devil tumours

**DOI:** 10.1111/eva.12117

**Published:** 2013-11-06

**Authors:** Beata Ujvari, Anne-Maree Pearse, Kate Swift, Pamela Hodson, Bobby Hua, Stephen Pyecroft, Robyn Taylor, Rodrigo Hamede, Menna Jones, Katherine Belov, Thomas Madsen

**Affiliations:** 1 Faculty of Veterinary Science, University of SydneySydney, NSW, Australia; 2 Animal Health Laboratory Department of Primary Industries, Parks and Water and Environment LauncestonTas., Australia; 3 School of Animal & Veterinary Science, Faculty of Science, University of AdelaideAdelaide, SA, Australia; 4 School of Zoology, University of TasmaniaHobart, Tas., Australia; 5 School of Biological Sciences, University of WollongongWollongong, NSW, Australia

**Keywords:** cancer evolution, genomic decay, Tasmanian Devil Facial Tumour Disease, tetraploidy

## Abstract

The Tasmanian Devil Facial Tumour Disease (DFTD) provides a unique opportunity to elucidate the long-term effects of natural and anthropogenic selection on cancer evolution. Since first observed in 1996, this transmissible cancer has caused local population declines by >90%. So far, four chromosomal DFTD variants (strains) have been described and karyotypic analyses of 253 tumours showed higher levels of tetraploidy in the oldest strain. We propose that increased ploidy in the oldest strain may have evolved in response to effects of genomic decay observed in asexually reproducing organisms. In this study, we focus on the evolutionary response of DFTD to a disease suppression trial. Tumours collected from devils subjected to the removal programme showed accelerated temporal evolution of tetraploidy compared with tumours from other populations where no increase in tetraploid tumours were observed. As ploidy significantly reduces tumour growth rate, we suggest that the disease suppression trial resulted in selection favouring slower growing tumours mediated by an increased level of tetraploidy. Our study reveals that DFTD has the capacity to rapidly respond to novel selective regimes and that disease eradication may result in novel tumour adaptations, which may further imperil the long-term survival of the world's largest carnivorous marsupial.

## Introduction

More than 35 years ago, Nowell (1976) suggested that cancer progression should be regarded as an evolutionary process. We now know that cancer is subjected to selective regimes similar to those experienced by asexually reproducing organisms (Merlo et al. 2006). Cancer cells, like other asexual organisms, do not undergo meiotic recombination. How tumour cells survive the loss of heterozygosity and the emergence of recessive mutations remains unresolved (Solé and Deisboeck 2004). Proposed mechanisms to counteract genomic decay include chromosomal rearrangements that alter normal cell cycles and apoptotic responses, chromosome breaks and tolerance of deleterious mutations (Merlo et al. 2010).

The presence of aberrant karyotypes in malignant cells was first observed over a century ago (von Hansemann 1890) and led to the recognition of the role of missegregating chromosomes in tumour development (Boveri 2008; Chow and Poon 2010). Indeed, most malignant tumours have been found to harbour structurally and numerically rearranged chromosomes and multiple centrosomes, the conjoint causes and consequences of abnormal mitosis (Sen 2000; Storchova and Pellman 2004; King 2008; Storchova and Kuffer 2008; Ganem et al. 2009; Little 2010; Mosieniak and Sikora 2010). Apart from segmental chromosome defects and single chromosome losses cancer cells exhibit altered ploidy, with chromosome numbers ranging from hypodiploid (i.e. having a chromosome number lower than the diploid number) to hypertetraploid (i.e. having a chromosome number greater than but not an exact multiple of the normal diploid number; for reviews see: Storchova and Pellman 2004; Storchova and Kuffer 2008; Davoli and de Lange 2011). Aneuploidy and polyploidy have, however, been shown to provide cancer cells with adaptive potentials (Yuen and Desai 2008). For example, polyploidization may provide adaptive advantage to cancer cells by masking deleterious mutations (chromosome losses, gene deletions and inactivating mutations) and ameliorating the effects of deleterious recessive alleles (Otto and Whitton 2000; Otto 2007; Davoli and de Lange 2011). Moreover, tetraploidy allows tumour cells to sustain a higher mutation rate and may stimulate additional genome structure modifications facilitating adaptive changes. The immediate effect of polyploidization is a general rise in cell volume, and slower development due to increased genome size (Cavalier-Smith 1978; Gregory 2001; Otto 2007). However, changes in ploidy also upset the geometric machinery used to segregate chromosomes resulting in unstable genomes, rapid genomic repatterning and increased genetic diversity (Wendel 2000; Otto 2007). The increased genetic polymorphism associated with tetraploidy may promote the survival of certain polyploid cells, stabilize their genomic configuration and therefore fuel the evolution of polyploid cell populations. Thereby, polyploidy not only appears to promote tumorigenesis, but also steers cancer cell progression through a fitness landscape during cancer evolution.

Although the evolution of neoplasm in human cancers occur on a timescale of years, anthropogenic selection, administered as medical treatments, has been shown to accelerate the development of novel and aggressive as well as drug resistant cancer strains (Merlo et al. 2010). A significant problem when investigating how such therapies may affect human cancer evolutionary trajectories is often the short lifespan of both tumours and patients. The Tasmanian Devil Facial Tumour Disease (DFTD) provides a unique opportunity to elucidate the long-term effects of natural and anthropogenic selection on cancer evolution. This contagious cancer was observed first in 1996 and is transmitted between Tasmanian devils (*Sarcophilus harrisii*) by biting during social interactions (Pearse and Swift 2006; McCallum et al. 2007; McCallum 2008; Murchison 2008). The disease generally results in death of infected animals within 6 months and has led to local extinctions of more than 90%, questioning the long-term survival of this iconic animal (Jones et al. 2007; McCallum 2008). Cytogenetic analyses have revealed that DFTD is caused by a rogue cell line (Pearse and Swift 2006), which originated from Schwann cells of the peripheral nerve sheath (Loh et al. 2006; Murchison et al. 2010). devil facial tumour (DFT) cells possess a highly rearranged genome, characterized by tumour-specific complex translocations and chromosomal rearrangements (Pearse and Swift 2006; Deakin et al. 2012; Pearse et al. 2012). The clonal nature of DFTs has been supported by both large-scale genomic (Miller et al. 2011; Murchison et al. 2012), immunohistological (Loh et al. 2006) and genetic analyses (Siddle et al. 2007, 2010; Belov 2011). Although four chromosomal variants (strains) have been observed, exhibiting minor cytogenetic differences, the genome of DFT cells appears to remarkably be stable (Deakin et al. 2012; Pearse et al. 2012).

In an attempt to reduce the prevalence of DFTD, infected devils, approximately 33%, were removed from one site, the Forestier Peninsula, in Tasmania between 2006 and 2010 (Lachish et al. 2010; Beeton and McCallum 2011). The disease eradication trial provides a unique opportunity to elucidate the long-term effects of anthropogenic selection on DFTD evolution. In this study, we use karyotypic analysis to investigate overall temporal changes of tumour ploidy. Furthermore, we investigate the possible effects of the removal programme on cancer evolutionary trajectories on the Forestier Peninsula compared with other areas of Tasmania not subjected to anthropogenic selection.

## Methods

### Samples

**Figure 1:**
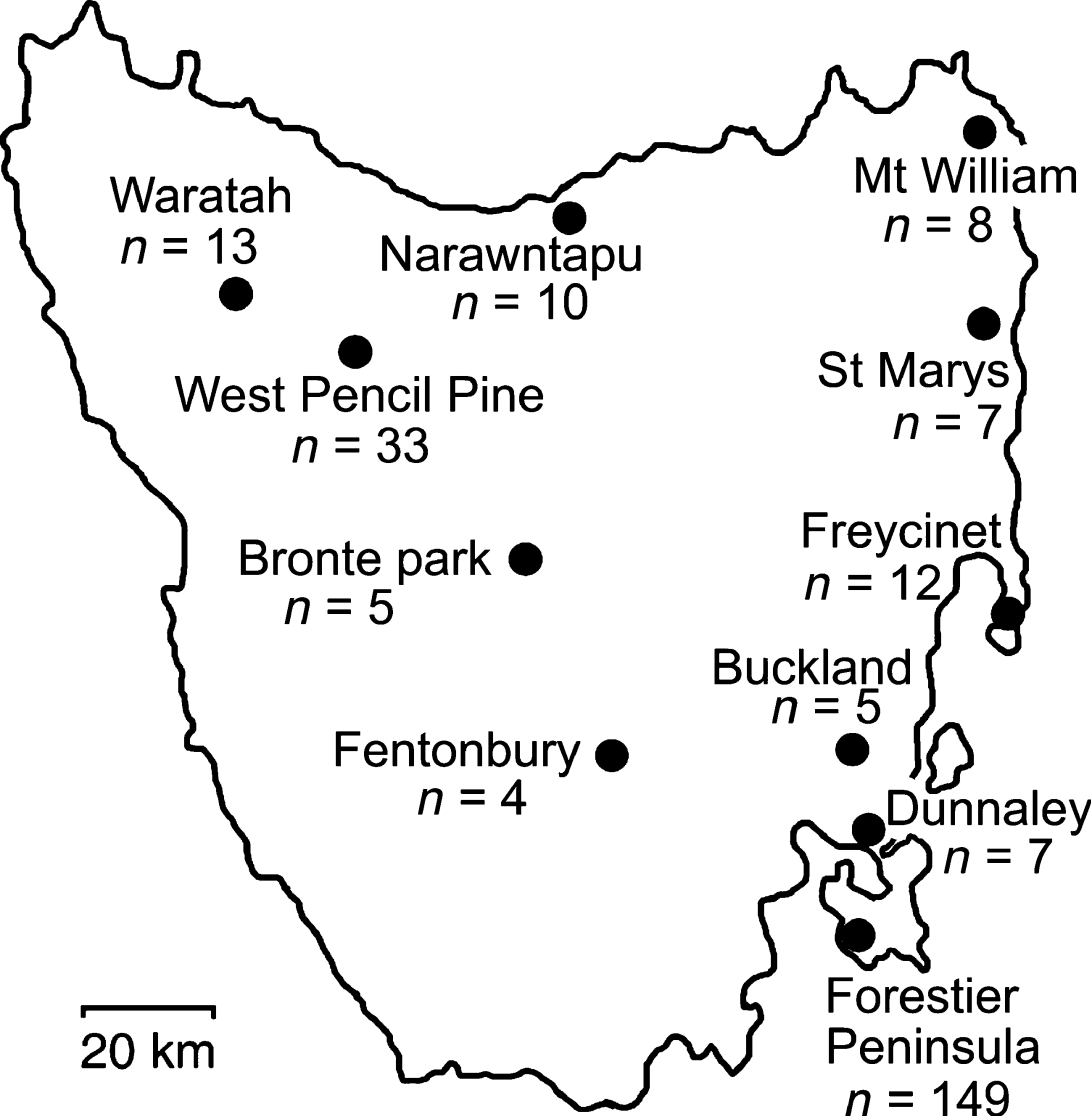
Map of Tasmania showing the location of the 11 sites sampled and number of samples collected at each location.

Tumour tissue samples used in the study were collected between 2006 and 2011 at 11 sites within the DFTD-affected areas of Tasmania (Fig. 1 which also provides data on number of samples collected at each of the 11 sites). A total of 253 diseased devils were analysed. Due to the trapping regimes employed, we were unable to obtain comparable number of samples from the 11 sites, making robust among-site comparisons unattainable. However, as the devil population on the Forestier Peninsula (Fig. 1) has been subjected to a disease suppression trial, that is, the removal of infected devils, we decided to investigate how such artificial selection may have affected tumour evolution. Of the 149 tumours collected at Forestier Peninsula, 148 were classified as strain 3 and one as strain 2. In contrast to most of the other sites, no samples were collected at Forestier Peninsula in 2011.

### Cell culture

Detailed description of DFTD cell culture and cytogenetic analysis has been previously described by Pearse et al. (2012). Briefly tissue biopsies and fine needle aspirates were transferred to sterile Petri dish and washed three times with Dulbecco's phosphate-buffered saline (Invitrogen, Carlsbad, CA, USA), with 0.1 mL penicillin/streptomycin solution (Sigma-Aldrich, St Louis, MO, USA) and 1 mg/mL amphotericin B (Sigma-Aldrich). Solid tissues were then disaggregated in 3 mL of prewarmed AmnioMax C-100 medium (Invitrogen/Life Technologies, Carlsbad, CA, USA) and homogenized in 3-mL syringe with an 18G needle until a milky single cell suspension was formed. Cancer cells were aliquoted into 20-mL culture flasks containing 8 mL of AmnioMax C-100 media (Invitrogen/Life Technologies), 0.1 mL of penicillin-streptomycin solution (Sigma-Aldrich), and 1 mg/mL of amphotericin B (Sigma-Aldrich). Cultures were incubated at 35°C. Tumour cells were harvested after 24–48 h in culture.

### Cytogenetic analysis

Three hours prior to harvesting 0.1 mL of demecolcine at 10 mg/mL (Sigma-Aldrich) was added to each culture. The cells were centrifuged for 10 min at 100 *g*. After the supernatant was discarded and the cell pellet was slowly resuspended in 7 mL of hypotonic 0.075 m KCl and placed in a water bath at 37°C for 10 min; 2 mL of chilled Carnoy's fixative (3:1 ratio of methanol and acetic acid) was added, and the tubes were centrifuged for 10 min at 100 *g*. The pellet was gently resuspended in fixative and stored at −2°C overnight. The following day, the cells were washed 4× in fresh fixative and resuspended. Chromosome spreads were achieved by adding a droplet of the suspension onto a frozen microscope slide. Slides were subsequently air-dried and incubated at 57°C for 3 days. G-banding was conducted by treating slides with a 0.15% solution of trypsin (Sigma-Aldrich) for 30 s, then staining with Leishman's stain for 2.5 min followed by mounting with Leica mounting medium (Leica Microsystems, North Ryde, NSW, Australia) for analysis. G-banding analysis was performed using a Leica DM 2000 microscope (Leica Microsystems) and photographed with a Leica DFC 420 C camera (Leica Microsystems). Karyotypes were made, originally by hand and later (from 2008) using Video testeKaryo 3.1 software (VideoTest, Saint Petersburg, Russia). At least 20 metaphases were analysed for each individual, and approximately, 200 cells were examined.

### Statistical analyses

Data were examined for normality before analysis, and when normality could not be achieved, nonparametric statistics were employed. Logistic regression was used when investigating temporal variation in tetraploidy among the 253 devil tumours where presence, that is, when all of 200 cells in the metaphases were recorded as being tetraploid, was entered as ‘1’ and total absence of tetraploids was entered as ‘0’. JMP, version 5.1 (SAS Institute Inc., Cary, NC, USA), were used in all statistical analyses.

## Results

**Figure 2:**
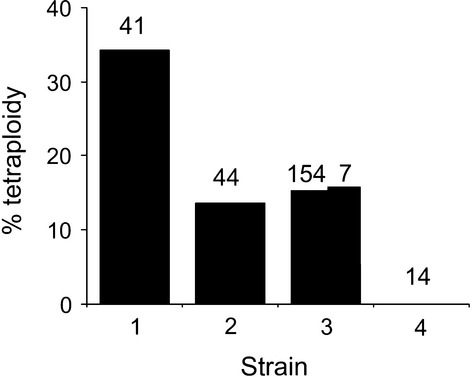
Proportion of tetraploid tumours recorded in the four DFTD strains. The numbers above the bars depict samples sizes. The two bars at strain 3 depict the proportion of tetraploid tumours when including (*n* = 154) and excluding (*n* = 7) the samples collected at Forestier Peninsula.

The large number of karyotypes used in this study made it possible to conduct robust tests to elucidate a possible variation in tetraploid tumours among the four strains. Our results revealed a significant variation of tetraploid tumours when employing the total number of samples collected at the 11 sites as well as when omitting the samples collected at the Forestier peninsula (χ^2^ = 11.7, *P* = 0.008, df = 3 and χ^2^ = 9.6, *P* = 0.02, df = 3, respectively, Fig. 2). Both analyses showed that the proportion of tetraploid tumours were highest in the strain 1, the oldest of the four strains (Fig. 2).

**Figure 3:**
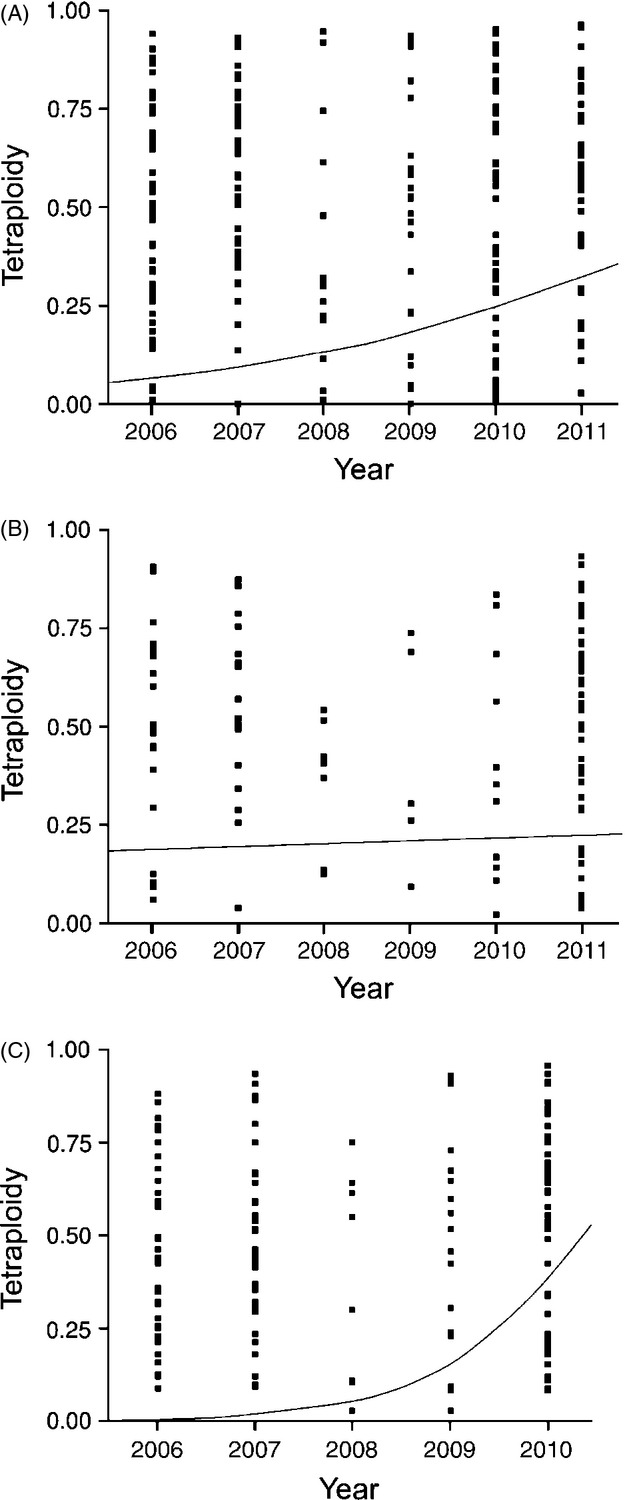
Logistic regression analyses of temporal variation in tetraploid tumours. Figure (A) Depicts the variation from 2006 to 2011 using our complete data set, that is, the analysis is based on all the 11 populations. Figure (B) Depicts the temporal variation in tetraploid tumours excluding the samples collected at Forestier Peninsula. Figure (C) Depicts the temporal variation in tetraploid tumours collected at the Forestier Peninsula.

We also observed a significant temporal increase in tetraploid tumours collected at the 11 sites from 2006 to 2011 (logistic regression with tetraploidy as dependent and year as independent variable: χ^2^ = 16.4, *P* < 0.0001, df = 1; Fig. 3A). However, when conducting the same analysis, but excluding the tumours collected from the Forestier Peninsula, and hence restricting the analysis to the 10 remaining sites, no temporal increase in tetraploid tumours was observed (χ^2^ = 0.15, *P* = 0.70, df = 1; Fig. 3B). A third logistic regression analysis, restricting the data to samples collected at Forestier Peninsula, revealed a significant temporal increase in tetraploid tumours among the Forestier devils (χ^2^ = 35.0, *P* < 0.0001, df = 1; Fig. 3C), clearly demonstrating that the overall temporal increase in tetraploid tumours was caused by the increase in tetraploids at the Forestier Peninsula.

## Discussion

Devil Facial Tumour Disease is a horizontally transferred asexually reproducing clonal cell line, which during the last 16 years have been exposed to the negative effects associated with Muller's ratchet, resulting in mutational meltdown and ultimately extinction. However, this obligate parasite has been able to survive and counteract the effect of deleterious mutations, genomic instability as well as being able to infect >100 000 devils (McCallum 2008). DFTD hence provides a unique opportunity to study cancer evolution *in vivo*.

Polyploidization is more common in asexual compared to sexual organisms (Otto and Whitton 2000) and provides an adaptive advantage to asexual organisms, such as cancer cells, by masking deleterious mutations and ameliorating the genomic decay process (Haldane 1933; Orr and Otto 1994; Orr 1995; Otto and Whitton 2000; Otto 2007; Davoli and de Lange 2011). The higher number of tetraploids recorded in the oldest DFT strain (strain 1) hence provides a possible additional mechanism by which this asexually reproducing obligate parasite has been able to avoid mutational decay. Increased ploidy has also been associated with slower tumour development and DFT cell growth rate (Pearse et al. 2012). During the disease suppression trial, at the appearance of first lesions, infected devils were removed from Forestier Peninsula (Lachish et al. 2010; Beeton and McCallum 2011). Such a selective regime could have favoured slower growing DFT cells ultimately resulting in the increased level tetraploid tumours observed at this location.

In a recent study, Murchison et al. (2012) suggested that the unique mitochondrial DFTD lineage present on the Forestier Peninsula had most likely emerged due to a selective sweep. Our results provide further evidence that the observed genetic and chromosomal changes at this site were most likely caused by selective sweep initiated by increased selection administrated via anthropogenic interaction (ongoing removal of DFTD infected devils). Unfortunately, the selective culling of infected devils neither slowed disease progression nor reduced population-level impacts of DFTD and was therefore abandoned in 2010 (Lachish et al. 2010).

Transition from whole-genome duplication via aneuploidy to malignancy is a common feature of several human cancers (for review see Davoli and de Lange 2011). In spite of reducing cell proliferation rates, tetraploidization has also been linked to the metastatic, aggressive as well as drug resistant stages of certain human malignancies (Castedo et al. 2006; Davoli and de Lange 2011). As suggested by Davoli and de Lange (2011), such significant changes in tumour phenotypes is most likely caused by an enhanced ability of tetraploid tumours to sustain a higher incidence of mutations, thereby increasing the probability of adaptive changes and increasing the probability that evolving tumours will accumulate mutations needed to progress to a malignant state. If the increased level of tetraploid tumours at Forestier Peninsula results in the evolution of a more malignant strain of DFTD, this may further imperil the long-term survival of the world's largest carnivorous marsupial. Although future studies are needed to elucidate the connection between malignancies and tetraploidy in DFTD, our study clearly demonstrates that DFTD tumours are able to rapidly respond to increased selection and adapt to a novel selective regime. Due to the observed low genomic (Miller et al. 2011; Murchison et al. 2012) and chromosomal polymorphism (Deakin et al. 2012; Pearse et al. 2012), DFT cells have been described as a stable, clonally evolving cell line. However, our recent studies show that this unique cancer is a dynamically evolving obligate parasite, which uses gene expression alterations (Ujvari et al. 2012, 2013), telomere homeostasis (Ujvari et al. 2012) and epigenetic variations (Ujvari et al. 2013). The results from the present study suggest that ploidization may offer yet another pathway to which DFTD is able to adapt to the ever-changing evolutionary landscape sculptured by the devils' immune system. Finally, our study is the first to show that anthropogenic selection may enhance cancer evolution in the wild, and it therefore cautions about what measures we employ to try to halt the spread of this devastating disease.

## Data archiving statement

Raw data for this manuscript are available in the Dryad Digital Repository: http://dx.doi.org/10.5061/dryad.327v0.
